# Diversity Analysis of the Rice False Smut Pathogen *Ustilaginoidea virens* in Southwest China

**DOI:** 10.3390/jof8111204

**Published:** 2022-11-15

**Authors:** Rongtao Fu, Cheng Chen, Jian Wang, Yao Liu, Liyu Zhao, Daihua Lu

**Affiliations:** 1 Institute of Plant Protection, Sichuan Academy of Agricultural Science, 20# Jingjusi Rd., Chengdu 610066, China; 2 Key Laboratory of Integrated Pest Management on Crops in Southwest, Ministry of Agriculture, Chengdu 610066, China; 3 Crop Research Institute, Sichuan Academy of Agricultural Science, Chengdu 610066, China

**Keywords:** rice false smut, *Ustilaginoidea virens*, diversity, morphology, pathogenicity, mating type, population structures

## Abstract

Rice false smut caused by *Ustilaginoidea virens* is a destructive disease in rice cropping areas of the world. The present study is focused on the morphology, pathogenicity, mating-type loci distribution, and genetic characterization of different isolates of *U. virens*. A total of 221 strains of *U. virens* were collected from 13 rice-growing regions in southwest China. The morphological features of these strains exhibited high diversity, and the pathogenicity of the smut fungus showed significant differentiation. There was no correlation between pathogenicity and sporulation. Mating-type locus (*MAT*) analysis revealed that all 221 isolates comprised heterothallic and homothallic forms, wherein 204 (92.31%) and 17 (7.69%) isolates belonged to heterothallic and homothallic mating types, respectively. Among 204 strains of heterothallic mating types, 62 (28.05%) contained *MAT1-1-1* idiomorphs, and 142 isolates (64.25%) had the *MAT1-2-1* idiomorph. Interestingly, strains isolated from the same fungus ball had different mating types. The genetic structure of the isolates was analyzed using simple sequence repeats (SSRs) and single-nucleotide polymorphisms (SNPs). All isolates were clustered into five genetic groups. The values of Nei’s gene diversity (*H*) and Shannon’s information index (*I*) indicated that all strains as a group had higher genetic diversity than strains from a single geographical population. The pairwise population fixation index (*F_ST_*) values also indicated significant genetic differentiation among all compared geographical populations. The analysis of molecular variation (AMOVA) indicated greater genetic variation within individual populations and less genetic variation among populations. The results showed that most of the strains were not clustered according to their geographical origin, showing the rich genetic diversity and the complex and diverse genetic background of *U. virens* in southwest China. These results should help to better understand the biological and genetic diversity of *U. virens* in southwest China and provide a theoretical basis for building effective management strategies.

## 1. Introduction

Rice false smut (RFS) caused by *Ustilaginoidea virens* (Cooke) Takah (teleomorph: *Villosiclava virens* [1] is one of the most destructive diseases of rice in the world. RFS was first reported in the Tirunelveli district of Tamil Nadu State, India, in 1878 [2]. The fungal pathogen infects rice flower organs and forms spore balls several times larger than rice seeds [3]. The surface of the spore balls is covered with a large number of chlamydospores that initially change from white to pale yellow, yellow, greenish-yellow, or greenish-black with time. Aside from the yield losses, RFS is also poisonous to humans and livestock, as the chlamydospores of *U. virens* produce large amounts of mycotoxins [4,5].

Understanding the morphological characterization of the pathogens will provide a theoretical basis to build optimal disease control strategies [6,7]. The *U. virens* from different geographical regions in different rice cultivars have produced various colony and chlamydospore characteristics in culture media [6,8]. The colony color in most of the strains of *U. virens* changes depending on maturity time, being initially white and changing to yellow and finally becoming green [9]. The rate of mycelial growth is variable, as the growth patterns range from very slow to slow, moderate, and fast [10]. The chlamydospores can be produced in culture media, and their sizes are significantly smaller than those produced in the field [6]. The spore surface is covered with spines visible at high magnification [8,11].

In ascomycetous fungi, mating-type (*MAT*) genes are the master loci that control sexual reproduction and development [12]. *MAT* genes have two idiomorphs, *MAT1-1* and *MAT1-2* [13]. Fungi can reproduce by selfing or outcrossing. In heterothallic ascomycetous fungi, each strain contains a single idiomorph, *MAT1-1* or *MAT1-2*, whereas homothallic fungi possess two idiomorphs [14]. Similar to other ascomycetes, sexual reproduction in *U. virens* is governed by the *MAT* loci [14,15]. Initially, a simple polymerase chain reaction (PCR)-based assay was used to identify the mating type of strains of *U. virens* [16]. Later, in our previous study [15], a multiplex PCR method was developed to detect the characteristics and distribution of *MAT1-1* and *MAT1-2* idiomorphs simultaneously. Although sexual reproduction in *U. virens* has been studied, the process remains poorly characterized. Mating type analysis is important to determine the molecular and genetic basis of sexual reproduction and the genetic variation of pathogens.

It is important to study the genetic population structure of plant pathogens for disease resistance breeding. In recent years, the development of molecular methods has promoted the research progress concerning the genetic diversity of plant pathogenic fungi. A large number of molecular markers have been explored and applied to the study of population genetic structure and genetic similarity of the fungi, including random amplified polymorphic DNA (RAPD), repetitive extragenic palindromic PCR (Rep-PCR), amplified fragment length polymorphism (AFLP), simple sequence repeats (SSRs), and single-nucleotide polymorphisms (SNPs) [17,18,19]. SSR markers are extensively applied in analyzing the genetic structure of the pathogens due to their presence in higher numbers, polymorphisms, better repeatability, codominance, and ubiquitous occurrence [20,21,22]. SNPs are third-generation molecular markers, as they depend on the analysis of sequence variation [19]. At present, SNPs are widely used for studying the genetic structure of various species due to their high density, genetic stability, and reliability of results [23,24].

At present, research on the population genetic structure of *U. viren* is becoming more mature, and a variety of molecular markers have been developed. Based on RAPDs and SSRs, Wang et al. [25] revealed that geographical environment played a greater role than rice cultivar in *U. virens* population selection. Sun et al. [26] explored the SNP markers of *U. virens* to analyze the genetic structure of *U. virens* from 15 provinces in China. Fang et al. [27] studied the genetic diversity of 167 *U. virens* strains from nine areas in Sichuan–Chongqing using SNP markers and found that gene flow occurred among different geographical populations. Furthermore, Tan et al. [7] used SNP markers to analyze the genetic variation of *U. virens* from the same rice varieties in Yunnan province, China. Bag et al. [10] studied the population genetic structure of 63 *U. virens* strains from seven states in eastern and northeastern India based on 17 RAPDs and 14 SSRs.

The southwest area (Sichuan province, Guizhou province, Yunnan province, and Chongqing, China) is one of the seven physical geographical subregions in China. It is one of the main rice planting areas due to the weather characteristics of rain and clouds, high humidity, less sunshine, and fertile soil. Rice false smut has become a very significant fungal disease of rice in this area. Currently, little information regarding the diversity characteristics of *U. virens* in the southwest area is available. Thus, this study investigated 221 strains of *U. virens* from 13 different rice-growing areas in southwest China for their morphological, pathogenic, and mating-type characteristics as well as their population genetic structure. The genetic structure was analyzed using SSR and SNP molecular markers. These results can be used to assist in managing the disease and also to develop false smut resistant varieties. These results can provide useful information for the occurrence regularity and comprehensive control of rice false smut disease.

## 2. Materials and Methods

### 2.1. Pathogen Strains and Culture Conditions

Naturally infected rice kernels showing representative yellow false smut symptoms were collected from 2017 to 2019 from 13 cities of southwest China, namely Bazhong (BZ), Chengdu (CD), Deyang (DY), Leshan (LS), Luzhou (LZ), Kunming (KM), Nanchong (NC), Lijiang (LJ), Yaan (YA), Yibin (YB), Chongqing (CQ), Guiyang (GY), and Zunyi (ZY) (Table 1). The pathogen *U. virens* was isolated from the yellow false smut balls. Initially, the smut balls were surface-sterilized using a UV lamp for 15 min on clean benches. Then, the chlamydospores on the surface of the balls were scattered on potato sucrose agar (PSA) plates. To avoid bacterial pollution, the medium was supplemented with chloramphenicol (100 ppm). Then, 100 µL of aseptic water was added to the plate and streaked on the plate to distribute the spores uniformly. The plates were incubated at 28 °C for 4 days. After 4 days of incubation, tiny white or yellow germinating spores were observed, and single colonies were transferred to fresh PSA medium for pure culture. A total of 221 strains were isolated in this experiment (Table 1).

To study the growth on PSA medium, 6 mm mycelium disks were placed into 150 mL flasks containing 100 mL potato sucrose (PS, made from a boiled extract of 300 g of peeled potatoes and 20 g of sucrose) fluid medium. The cultures were incubated at 28 °C on a shaker at 150 rpm for 14 days. The hyphae and conidia were collected by filtration and centrifugation, respectively. The mycelia were resuspended in the PS and pulverized for five minutes with a crusher. The conidia were added back to the hyphae fragment suspension to produce a mixture of spores and hyphae fragments used for inoculation.

### 2.2. Morphological Characterization of Strains of U. virens

All isolates were grown on PSA, and their cultural characteristics (colony color, diameter, and growth type) were observed. Chlamydospores produced on the PSA were characterized separately by scanning electron microscopy (SEM). The specimens for SEM were prepared according to the methods of Fu et al. [8] and Hu et al. [28], with minor modifications. The SEM samples were fixed for 2–4 h in TBS (0.05 M Tris–HCl, 0.15 M NaCl, pH 7.0) containing 2.0% glutaraldehyde. They were then dehydrated in a graded series of alcohol for 15 min each and prepared for critical point drying in carbon dioxide. After the samples were coated with a thin layer of gold, they were observed by scanning electron microscopy (JSM-5900LV; JEOL Ltd., Tokyo, Japan).

### 2.3. Plant Materials and Inoculation

A conventional rice variety, *Oryza sativa* L. spp. indica variety 93-11, was used in this study. The rice plants were grown in an air-conditioned greenhouse, with the temperatures ranging from 20 °C at night to a maximum of 36 °C during the day. The inoculation protocols described by Hu et al. [28] and Fu et al. [29] were used with some modifications. Approximately 2 mL of a hyphae fragment suspension was injected into leaf sheaths at the middle site on panicles at the seventh to eighth stage of panicle development [30]. The controls were injected with sterile PS for all experiments. Each treatment consisted of three parallel biological replicates, and each replicate comprised a pool of 25 panicles. After inoculation, all rice plants were kept at 25/30 °C (night/day), covered with a sunshade net, and automatically sprayed with water every 2 h for 10 min to maintain an environment with 90–95% relative humidity (RH). After 4 days, the sunshade net was uncovered, and the rice plants were grown under the normal greenhouse conditions at 25–35 °C and 80–100% RH.

### 2.4. Correlation Analysis between Pathogenicity and Sporulation of U. virens

The conidia of pathogenicity-tested strains were counted using a hemocytometer (Shanghai Qiujing Biochemical Reagent Instrument Co., Ltd., Shanghai, China). SPSS 19.0 analysis software was used to analyze the correlation between pathogenicity and sporulation of the strains and to draw scatter plots. The correlation degree was analyzed according to the correlation coefficient (*R*), with *R* < 0 indicating a negative correlation, *R* = 0 indicating no linear correlation, |*R*| > 0.95 defined as a significant correlation, |*R*| > 0.8 defined as being highly relevant, 0.5 ≤ |*R*| < 0.8 indicating a moderate correlation, 0.3 ≤ |*R*| < 0.5 indicating a low correlation, and |*R*| < 0.3 considered irrelevant [9,31]. A significance probability *p* < 0.05 indicated that the difference was statistically significant.

### 2.5. Genomic DNA Extraction

Each isolate was inoculated into 100 mL PS liquid medium for 10 days at 28 °C on a 150 rpm incubator shaker. The hyphae of each isolate were harvested by filtration and immediately ground to a powder with liquid nitrogen for genomic DNA extraction. The DNA was extracted using a modified cetyl trimethyl ammonium bromide (CTAB) method [32]. The quality and quantity of the isolated DNA were estimated by 1% agarose gels and an Agilent 2100 Bioanalyzer (Agilent Technologies, Santa Clara, CA, USA). The DNA samples were diluted into a working solution and stored at −20 °C for future use.

### 2.6. Mating-Type Analysis

Two primer sets, MAT1-1F/MAT1-1R and MAT1-2F/MAT1-2R (Table 2), were used to detect the mating type of *MAT1-1-1* and *MAT1-2-1* in isolates of *U. virens*, respectively [14]. The multiplex PCR was used to analyze the mating-type of *U. virens*. The PCR reaction mixture (20 μL) included 10 μL of Goldenstar T6 super mix (TsingKe, Beijing, China), 0.2 μM of each primer (MAT1-1F/MAT1-1R/MAT1-2F/MAT1-2R), 4 μL of nuclease-free water, and 10 ng of genomic DNA of *U. virens*. The PCR products were amplified according to the following conditions: initial denaturation at 94 °C for 2 min, followed by 35 cycles of denaturation at 94 °C for 30 s, annealing at 60 °C for 40 s, extension at 72 °C for 40 s, and a final elongation step for 5 min at 72 °C.

### 2.7. SSR Analysis

Out of 17 SSR primers, nine primers produced polymorphisms among the tested strains in preliminary experiments (Table 2). PCR was performed in a 20 μL reaction volume that contained 10 μL of Goldenstar T6 super mix (TsingKe, Beijing, China), 10 μM of each primer, 1 μL of template DNA (approximately 10 ng), and 7 μL of nuclease-free water. Amplification was performed as described by Yu et al. [22] in a thermal cycler (Analytica Jena GMBH, Jena, Thuringia, Germany) with the following program: initial denaturation at 94 °C for 3 min, followed by 35 cycles of denaturation at 94 °C for 20 s, annealing at 56 °C for 30 s, elongation at 72 °C for 1 min, and a final extension at 72 °C for 5 min. The PCR products were isolated on an 8% (*w*/*v*) polyacrylamide gel [33].

Polymorphic DNA bands were manually scored as binary data, with the presence and absence of bands scored as 1 and 0, respectively. The polymorphism data were entered into the software package Numerical Taxonomy Multivariate Analysis System (NTSYS-pc), version 2.10 (Department of Ecology and Evolution, State University of New York) to establish a dendrogram using the unweighted pair-group method with arithmetic means (UPGMA) algorithm in the SAHN program.

### 2.8. SNP Analysis

Based on the genomic sequence of *U. virens*, three SNP primer pairs that could amplify SNP-rich regions were used in this study (Table 2) [26]. The PCRs were performed in a total reaction volume of 40 μL containing 20 μL of Goldenstar T6 super mix (TsingKe, Beijing, China), 0.2 μM of each primer, 40 ng of template DNA, and 14 μL of nuclease-free water. PCR was performed as described by Sun et al. [26] in a thermal cycler (EasyCycler, Germany) with the following profile: initial denaturation at 94 °C for 2 min, followed by 35 cycles of denaturation at 94 °C for 20 s, annealing at 56 °C for 30 s, elongation at 72 °C for 1 min, and a final extension at 72 °C for 5 min. Amplified PCR products were isolated by electrophoresis and purified for sequencing.

Sequencing was conducted at Tsingke Biotechnology Co., Ltd. (Chengdu, China) and aligned using the program Cluster W in the software MEGA 5.2 [34]. The neighbor-joining (NJ) method was applied to establish phylogenetic trees. Bootstrap tests were based on 500 resamplings, and midpoint rooting was used for each dataset.

### 2.9. Population Genetic Analysis

Population genetic analysis was carried out within and between populations using SSR and SNP data. For the cross-population analysis, *F_ST_* values were computed using Arlequin 3.5 based on SSR data and DNA sequences [35]. Principal coordinate analysis (PCoA) was performed with individual strains using GenALEx v.6.5 [36]. The population structure was analyzed with the help of SSR and SNP data using STRUCTURE v.2.3.4 [37]. The number of clusters (delta *K*) was computed using an ad hoc statistical method with different *K* values from 1 to 14 and with five independent repetitions per *K* value along with a 10,000 burn-in period and 100,000 Markov chain Monte Carlo (MCMC) iterations [10,37]. The peak value of delta *K* was determined using STRUCTURE HARVESTER (available at http://taylor0.biology.ucla.edu/structureHarvester/, accessed on 1 August 2022), and this was considered to be the optimal number of genetic groups [38]. A Mantel test was carried out to evaluate the correlation between genetic distance and geographic distance using GenALEx v.6.5. Analysis of molecular variance (AMOVA) tests were carried out to evaluate population variance among and within populations using GenALEx 6.5 based on the DNA sequences and SSR data.

## 3. Results

### 3.1. Isolation and Morphological Features of U. virens

**Figure 1:**
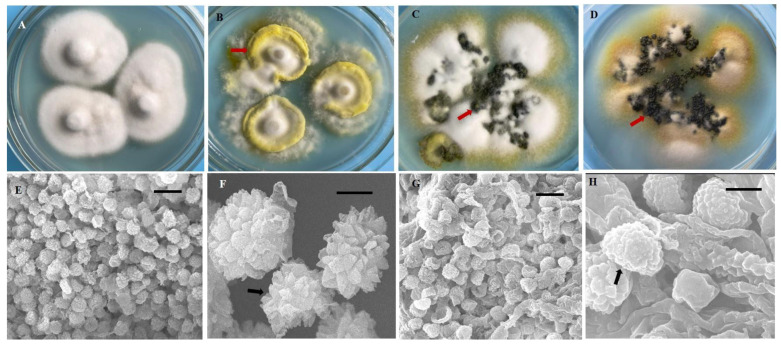
Diversity in morphology and chlamydospores of *Ustilaginoidea virens* strains. (**A**) Morphology of the colony resembled a straw hat at 21 d after inoculation. (**B**–**D**) There were many mounds of chlamydospores (arrows) formed on the colony margin and at the center. The red arrows indicate the colors (**B**) yellow, (**C**) greenish-black, and (**D**) black. (**E**–**H**) Ultramorphological characteristics of *U. virens* chlamydospores from natural false smuts and laboratory cultures. (**E**,**F**) Natural false smut. (**G**,**H**) Laboratory culture. Bars: (**E**,**G**) = 10 μm. (**F**,**H**) = 1 μm.

In this study, we obtained 221 strains of *U. virens* from 13 rice cropping regions in southwest China (Table 1). These isolates were grown on PSA medium, and their morphological features exhibited significant diversity. After 21 days, most isolates (*n* = 127) formed yellow or pale yellow-colored colonies, whereas 44 isolates formed white or cream-white colonies, 36 isolates formed greenish-yellow colonies, and 14 isolates formed black colonies (Supplementary Table S1, Figure 1A–D). The mycelial growth rate of the isolates was variable (growth rate from 1.06 to 2.24 mm/d) (Supplementary Table S1), as their growth patterns varied from very slow to slow, moderate, and fast. All isolates produced chlamydospores on PSA medium. The chlamydospores were formed at the center or the margin of the colony. The chlamydospores produced in the PSA medium and those produced on smut balls in the field were compared. The chlamydospores produced in the PSA were smaller than those produced on the smut balls. The mean diameter of the chlamydospores produced in the PSA was 0.76–0.95 µm, whereas this was 1.01–1.33 µm for the field chlamydospores. The color of chlamydospores formed on smut balls was yellow, orange, greenish-black, or black, whereas only three different colored types (yellow, greenish-black, and black) were observed in the PSA chlamydospores (Figure 1B–D). The chlamydospores were observed to have three different shapes, being circular, circular irregular, or circular oval in PSA and in the field. The chlamydospores clearly displayed prominent spore ornamentations under higher magnifications of scanning electron microscopy, previously referred to as processed spines [11]. The spines of natural chlamydospores were pointed at the apex or were irregularly shaped (Figure 1E,F), whereas those formed on the PSA were arc-shaped (Figure 1G,H).

### 3.2. Pathogenicity Determination of Isolates in Different Rice-Cropping Regions

The pathogenicity of 104 isolates from 13 rice-cropping regions was determined in this experiment. The rice panicles inoculated with *U. virens* were observed after 21 dpi. The diseased grains from each panicle were investigated, and the data were subjected to statistical analysis (Supplementary Table S1). The results showed that the pathogenicity of strains isolated from the same or different areas of the rice panicle differed, and the pathogenicity rate and average number of infected grains were 0%–100% and 0–69.64, respectively. Among the 104 isolates, the pathogenicity of 39 (17.65%) isolates was greater than 60%, referred to as strongly virulent strains [9,31]. Among the rice-cropping regions, 22 (56.41%) out of 39 isolates were from Chengdu, and three (7.69%) were from Luzhou and Zunyi; the rest were from Bazhong, Deyang, Kunming, Nanchong, Yaan, and Chongqing. In addition, the pathogenicity of the strains isolated from the same smut balls and panicle varied among the rice panicles. Therefore, the results revealed differentiation in the pathogenicity of strains of *U. virens*, but there was no clear geographical pattern.

### 3.3. Correlation between Pathogenicity and Sporulation

**Figure 2:**
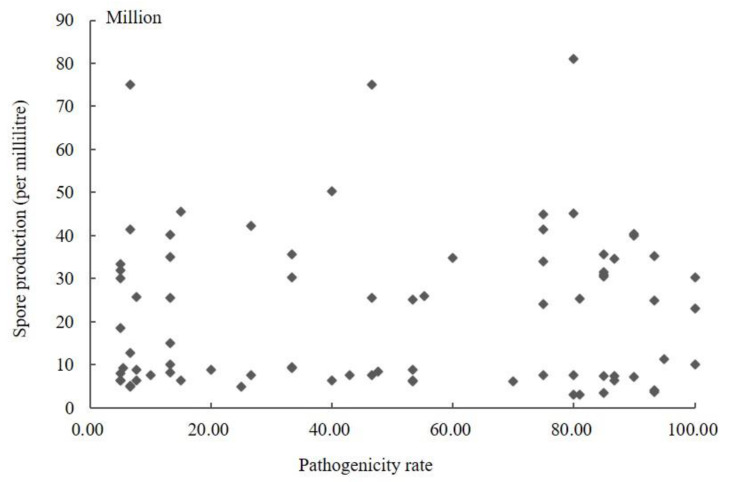
Scatter plot of sporulation quantity and pathogenicity of *Ustilaginoidea virens*.

The conidia produced by 104 isolates in PS were counted using a hemocytometer (Supplementary Table S1). The results showed that the sporulation quantity of the different strains varied, ranging from 3.00 × 10^6^ to 8.77 × 10^8^. The highest spore yield was 292.33 times higher than the lowest. The amount of sporulation of strains was not associated with stronger pathogenicity. The conidia yield of the strains with a pathogenicity rate greater than 60% was 3.00 × 10^6^–4.56 × 10^8^, and the pathogenicity rate of the strains with sporulation greater than 8.00 × 10^8^ was less than 13.50%. The scatter plot of sporulation and pathogenicity of 104 strains was obtained using SPSS 19.0 software (Figure 2). It can be seen from the figure that the correlation coefficient *R* was 0.258 (*p* = 0.106); so, the correlation was not statistically significant. Therefore, these results indicated no significant correlation between the pathogenicity of the fungus and its conidia production.

### 3.4. Mating-Type Locus Analysis

**Figure 3:**
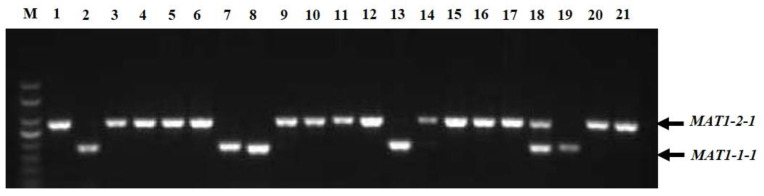
Multiplex PCR amplification of mating-type genes from *Ustilaginoidea virens* isolates. Lane M, DNA marker (Trans2K, TransGen); Lanes 1–21 represent isolates CDQ1-1, CDQ2-1, KMJ1-1, CDP1-1, BZN1-1, DYG1, CDX1-1, CDX4-1, CDX2-1-1, CDX5-1, LZG1-1, CDX3-1, CDPJ1, CDPJ2, KMJ2-1, LZD1, CDJ1-1, LZG1-2, CDC1, YAZ1, and CDPJ3, respectively.

In the multiplex PCR assay using idiomorph-specific primers, the *MAT1-1-1* and *MAT1-2-1* idiomorphs were successfully amplified (Figure 3). The genomic DNA of all 221 strains was subjected to multiplex PCR with MAT1-1F/MAT1-1R and MAT1-2F/MAT1-2R primer pairs that amplified 185 bp of the *MAT1-1-1* idiomorphs and 285 bp of the *MAT1-2-1* idiomorphs. Among 221 strains, 62 (28.05%) isolates contained *MAT1-1-1* idiomorphs and were classified as *MAT1-1-1* heterothallic mating type, 142 strains (64.25%) had the *MAT1-2-1* idiomorph and were classified as *MAT1-2-1* heterothallic mating type, and 17 isolates (7.69%) contained both *MAT1-1-1* and *MAT1-2-1* idiomorphs and were classified as the homothallic mating type (Table 1). Among the cities, Chengdu, Deyang, Luzhou, Lijiang, Yaan, and Chongqing contained not only heterothallic mating-type isolates with *MAT1-1-1* or *MAT1-2-1* idiomorphs, but also homothallic mating-type strains with both *MAT1-1-1* and *MAT1-2-1* idiomorphs. In Kunming, Lijiang, Yaan, and Zunyi, the mating types of the strains were mainly *MAT1-1-1* and *MAT1-2-1* heterothallic mating types, whereas in Bazhong and Guiyang, the isolates were only a *MAT1-2-1* idiomorph heterothallic mating type. Interestingly, strains isolated from the same ball had different mating types; for example, strains CQN1-1-1 and CQN1-1-2 had the *MAT1-2-1* idiomorph, whereas strains CQN1-1-3, CQN1-1-4, and CQN1-1-5 had the *MAT1-1-1* idiomorph.

### 3.5. Phylogenetic Analysis of Strains Based on SSR Molecular Markers

SSR analyses were performed with 221 strains of *U. virens* from 13 cities in southwest China. With nine SSR primers, 40 unambiguous bands were amplified. Of these, 25 were polymorphic, accounting for 62.50% of the polymorphism. The number of SSR alleles varied from 3 to 8. The average number of bands amplified per SSR primer was 4.4. The polymorphism of primer RM211 was the highest, and the polymorphism band ratio reached 75%. RM509 and RM523 showed low polymorphism, and the polymorphism band ratio was only 33.33% (Table 3).

**Figure 4:**
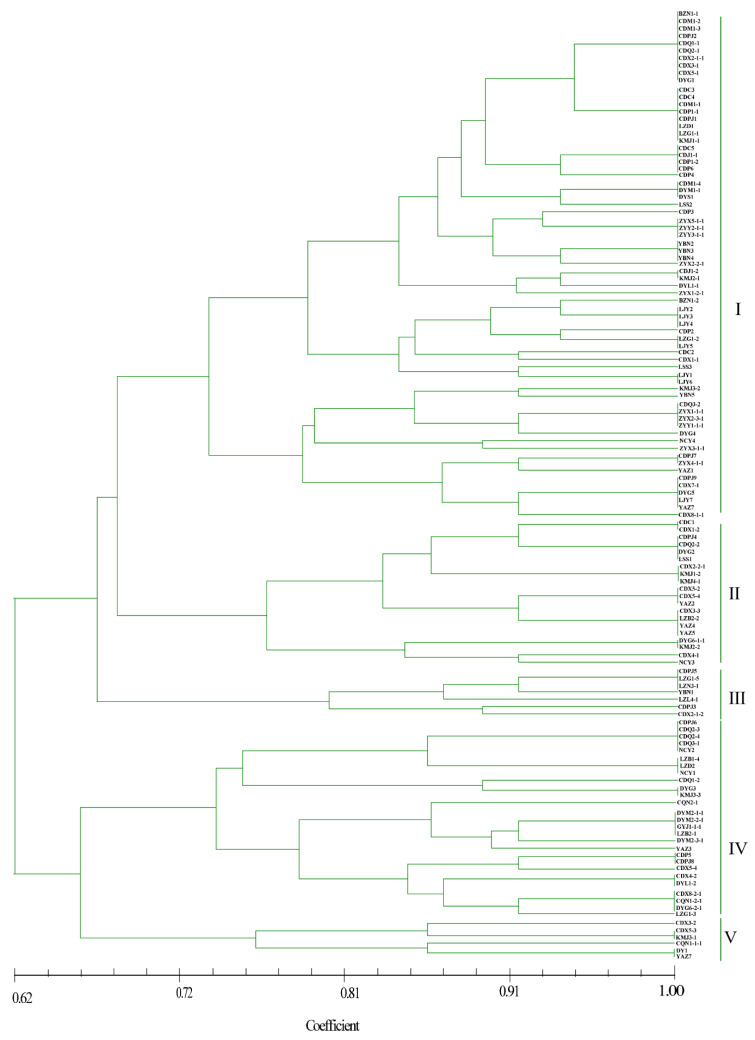
Dendrogram constructed with the unweighted pair-group method with arithmetic means (UPGMA) clustering method for isolates of *Ustilaginoidea virens* based on nine SSR primers. Five groups were divided at 0.72 genetic distance level, and most isolates were in groups I and IV.

As shown in Figure 4, most isolates from the BZ, DY, CQ, GY, and ZY regions grouped together, whereas strains from the other eight locations did not. Five genetic groups were divided at a 0.72 genetic distance level: 52.13% of the isolates were classified into group I, including BZ, CD, DY, LJ, and ZY, and 16.81%, 5.88%, 20.17%, and 4.20% of the isolates were classified into groups II, III, IV, and V, respectively (Figure 4 and Supplementary Figure S1). The identity of some strains from the same field, but from different smut balls, reached 100%, such as in CDM1-2, CDM1-3, CDPJ2, CDQ1-1, CDQ2-1, CDX2-1-1, CDX3-1, and CDX5-1; CDC5, CDJ1-1, CDP1-2, and CDP6; and ZYX5-1-1, ZYY2-1-1, and ZYY3-1-1 (Figure 4). In contrast, the isolates DYM2-1-1, DYM2-1-2, DYM2-1-3, DYM2-1-4, and DYM2-1-5 obtained from the same smut balls showed 100% identity, and CDX8-1-1, CDX8-1-2 CDX8-1-3 CDX8-1-4 CDX8-1-5, and CDX8-1-6 from the same smut balls showed high similarity. Other strains from the same smut balls were not in the same subgroups (Supplementary Figure S2).

As shown in Table 4, the percentage of polymorphic loci for all strains (86.13%) was higher than those for geographical populations, which varied from 53.08% to 85.67%. Similarly, the numbers of different alleles (*Na*) (1.8751) and effective alleles (*Ne*) (1.4231) for all isolates were higher than those for geographical populations, where the values ranged from 1.1034 to 1.8165 and from 1.056 to 1.4017, respectively. Nei’s genetic diversity (*H*) and Shannon’s information index (*I*) for all strains were 0.2994 and 0.4016, and for geographical populations, the values ranged from 0.1701 to 0.2893 and from 0.2543 to 0.3856, respectively. The results showed that the values of *H* and *I* revealed similar tendencies in that all strains as a group had higher genetic diversity than strains from individual geographical populations.

### 3.6. Phylogenetic Analysis of Strains Based on SNP Markers

**Figure 5:**
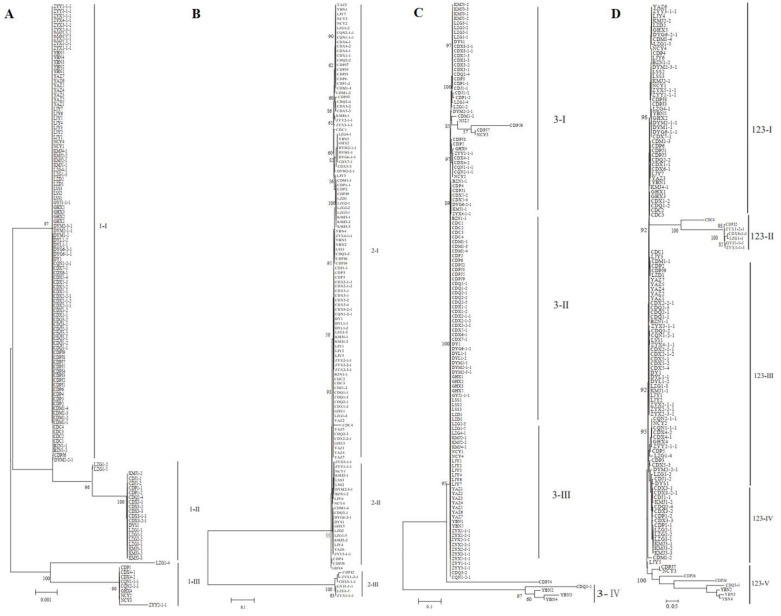
Phylogenetic analysis based on SNP sequence at individual markers (**A**–**C**) or the combined SNP sequence at three markers (**D**). NJ phylogenetic analyses were conducted based on DNA sequences at marker 1 (**A**), marker 2 (**B**), and marker 3 (**C**) and the combined sequences at three markers (**D**). Nodal support values for NJ are given for branches receiving more than 70% support. Groups were named with marker names and Roman numerals.

The 221 strains from 13 fields in southwest China were phylogenetically analyzed using three SNP molecular markers. As shown in Figure 5A–C and Supplementary Figure S3A–C, the strains could be divided into three, three, and four groups according to DNA sequences at marker 1, marker 2, and marker 3, respectively. Most of the groups included strains from different rice-growing areas. All isolates could be clustered into five genetic groups according to the combined DNA sequences at the three SNP molecular markers (Figure 5D and Supplementary Figure S3D). In addition, the results showed that some isolates from the same rice false smut balls were divided into different groups (Supplementary Figure S4).

As shown in Figure 5D, the isolates were divided into five genetic groups: 51.94% of the isolates were classified into group III, including CD, DY, LJ, and ZY, whereas 36.43%, 5.43%, 12.40%, and 6.20% of the isolates were classified into groups I, II, IV, and V, respectively. The group classification using SNPs was similar to the SSR analysis. CDM1-3, CDP6, CDPJ1, CDPJ5, CDQ2-2, CDX1-1, and CDX6-1 from different false smuts in the Chengdu field were clustered together, and YAZ1, YAZ2, YAZ4, YAZ5, and YAZ7 from different false smuts in the Yaan field were in the same clade (Figure 5D). The strains DYM2-1-1, DYM2-1-2, DYM2-1-3, DYM2-1-4, and DYM2-1-5 obtained from the same false smut in the Deyang field shared 100% identity, while ZYX2-1-1, ZYX2-1-2, ZYX2-1-3, ZYX2-1-4, ZYX2-1-5, and ZYX2-1-6 from the same false smut shared 100% identity (Supplementary Figure S4). Other strains from the same false smut were distributed in different clades.

### 3.7. Population Genetic Structure

**Figure 6:**
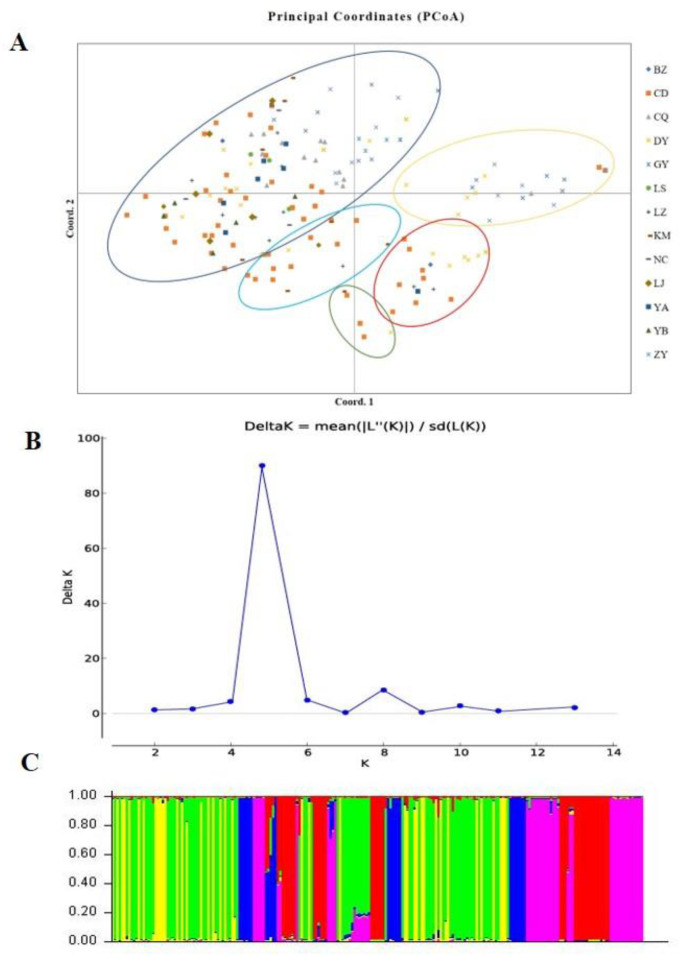
Population structure analysis of *Ustilaginoidea virens* isolates based on SSR data. (**A**). Principal coordinate analysis. (**B**). Line chart of change in K value. (**C**). Population structure at K = 5.

According to the SSR and SNP data, the two different populations were compared, and the *F_ST_* values indicated that significant genetic differentiation could be found in most populations, except for the NC versus BZ, DY versus CD, and LZ versus YA comparisons (Supplementary Table S2). However, the two methods also indicated minor inconsistencies (LZ versus LS and YB versus YA). According to the principle that close location indicates a close relationship and distant location indicates a distant relationship, a PCoA of all isolates was conducted (Figure 6A). The scatter plot from the PCoA showed that the first two axes contributed 12.73% and 10.49% variation, respectively. As shown in Figure 6A, all strains were divided into five genetic groups, and the location of the isolates of each group was complex, a result that was similar to phylogenetic clustering analysis. The PCoA showed that *U. virens* strains were not clustered according to their origin.

**Figure 7:**
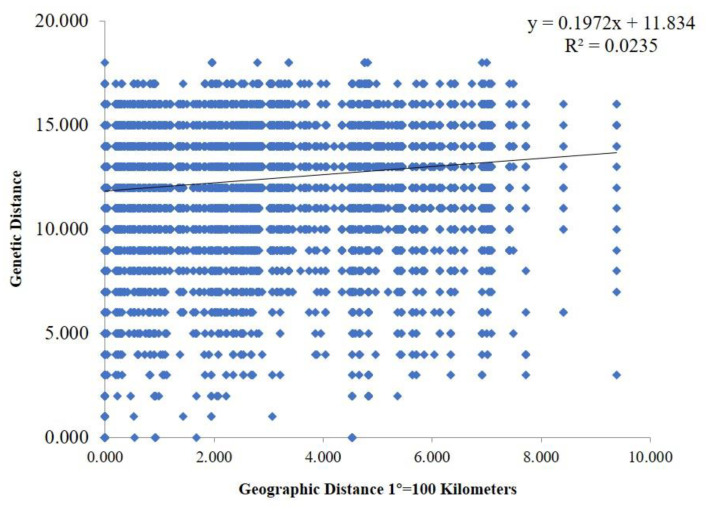
Mantel test between genetic distance and geographic distance. The geographic distance between different areas was calculated based on their corresponding longitude and latitude.

Genetic differences among strains within populations between different populations were also confirmed via model-based clustering algorithm analysis by STRUCTURE v 2.3.4. According to the principle of maximum likelihood value, the appropriate *K* value was selected as the number of populations [39]. As shown in Figure 6B, five was the optimal number of populations. Therefore, all isolates could be divided into five groups according to their genetic structure (Figure 6C). Combined with the location of each isolate, it was found that the isolates were not classified according to geographical origin, and the results of the analysis were basically consistent with the results of the PCoA. A Mantel test was used to assess the relationship between the genetic distance and geographic distance among populations, and *R*^2^ = 0.0235 (*p* = 0.15) in the regression equation, showing no significant correlation between the genetic distance and geographic distance (Figure 7).

### 3.8. AMOVA

AMOVA was performed by separating the total variation among populations within populations and among isolates within populations. The AMOVA showed that the difference between these populations contributed 21% of the total genetic variation, while 79% of the genetic variation came from within individual populations (Supplementary Figure S5). These results indicated that the individual geographical populations had rich genetic diversity.

## 4. Discussion

RFS is one of the main devastating diseases in the rice-planting areas of the world, seriously affecting the yield and quality of rice. Most studies have focused on infection processes [28,40], mycotoxins [41,42], or pathogenic factors [29,43] in the causal agent of RFS, *U. virens*. However, a systematic study on the biological characteristics and genetic structure are useful for improving the strategies for disease management and resistance breeding; yet, very little is known concerning these topics. In view of this, the current study aimed to investigate the morphology, pathogenicity, mating-type loci distribution, and population structure of the pathogen.

The study on the morphology and growth characteristics of *U. virens* was significant for further understanding the occurrence regularity of RFS and control measures against RFS [6]. In China, the morphology and growth characteristics of *U. virens* have been reported by some researchers [8,44,45]; however, the few isolates considered came from limited geographical regions. In this study, a total of 221 strains of *U. virens* from 13 rice-growing areas of southwest China were collected. All strains were cultured on PSA medium, and their colonies and chlamydospore morphological characteristics exhibited significant diversity. The growth rate patterns of the mycelium in the medium varied from very slow to slow, moderate, and fast. The variable growth pattern of *U. virens* mycelia was consistent with previous reports [10]. At high magnification, this study identified the morphological differences between artificially reared and natural chlamydospores, showing clear differences on the surface of the spores. The cultured chlamydospores were shown to have smooth and regular spines; however, the natural chlamydospores had sharp or irregular spines. In addition, the size of cultured chlamydospores was smaller than that of natural chlamydospores. These results were generally consistent with those reported in our previous study [8]. However, our results differed from those of Sharanabasav et al. [6]. Here, we reported the diversity of colony morphology and the growth characteristics of geographically distinct isolates from southwest China; however, there was no correlation between these characteristics of strains and their geographical origin.

The pathogenicity of *U. virens* from different geographical sources varied, but there was no clear geographical division [9,46]. Li et al. [46] determined the virulence of 51 strains of *U. virens* from eight cities of Liaoning Province, and the results showed that the virulence of the isolates from different geographical sources varied; the same pathogenic type of strains belonged to different genetic groups, and the same genetic group contained different pathogenic strains. In this experiment, we determined the pathogenicity of 104 isolates form 13 rice-cropping regions. The results indicated that the pathogenicity of the isolates of *U. virens* from different origins varied; even the pathogenicity of the strains isolated from the same smut ball and panicle varied among rice panicles, but there was no clear geographical division. Therefore, this study is consistent with previous research reports [46,47]. In addition, this study is the first to report that there was no significant correlation between the pathogenicity of the fungus and its conidia production.

Fungal *MAT* genes play a vital role in regulating sexual reproduction, virulence, and survival. The mating type of fungi can be divided into homothallism and heterothallism [13]. The sexual reproduction of *U. virens* is also controlled by *MAT* loci [14,15]. Previously, it was reported that *U. virens* was only homothallic or heterothallic [6,15,16]. In the present study, previously published *MAT1-1* and *MAT1-2* loci-specific primers [14] were applied to identify the *MAT* loci of 221 strains from 13 rice-growing regions of southwest China. We determined that the southwest China *U. virens* population contains heterothallic and homothallic isolates. Among the heterothallic isolates, 62 isolates contained *MAT1-1-1* idiomorphs, and 142 isolates had the *MAT1-2-1* idiomorph. This result differs from those reported by Fu et al. [15] and Yu et al. [14], who found that *U. virens* was only homothallic or heterothallic. This difference may be due to some isolates coming from limited geographical regions. Previous studies have reported that the mating types of some filamentous fungi are only heterothallic or homothallic [48,49]. The present study found that *U. virens* contained both heterothallic and homothallic strains. In addition, we found an interesting phenomenon, i.e., the strains isolated from the same ball had different mating types.

AFLP, SSR, RAPD, and SNP molecular markers have been widely applied to genetic diversity studies of *U. virens* [10,17,25,27]. Compared with other molecular markers, SSRs and SNPs are more unambiguous, stable, accurate, and reliable for evaluating population genetic structure [10,25]. In view of this, SSRs and SNPs were employed to confirm genetic diversity among different strains of *U. virens* from 13 rice-growing regions of southwest China to verify whether population structure was related to geographical origin. The phylogenetic analyses accorded to SSRs and SNPs were generally in agreement. They both showed that the 221 strains from 13 rice-growing fields were divided into five genetic groups, and most strains from the same field, but from different smut balls, tended to cluster together, as verified by PCoA and population structure analyses. Most isolates from BZ, DY, CQ, GY, and ZY regions clustered together, whereas the strains from the other locations did not. Similar to a previous report by Bag et al. [10], no location-specific clustering was observed in this study. The values of Nei’s gene diversity (*H*) and Shannon’s information index (*I*) indicated that all strains as a group had higher genetic diversity than strains from individual geographical populations. The findings were consistent with the study of Wang et al. [25].

To date, the correlation between the genetic variation of *U. virens* and geographical environment has been widely studied and discussed with some controversy. Zhang et al. [50] found that the strains within lineages were not correlated with sample origins. Yang et al. [51] pointed out that the clustering of isolates had no clear relationship with their geographical location. However, Fang et al. [27] found a high degree of genetic variation of *U. virens* among geographical populations. In this study, the pairwise *F*_ST_ values showed that most populations showed significant genetic differentiation. This is consistent with the conclusion that geographical environment has a vital influence on the fungal population. Because the 13 areas are in the southwest region with abundant rainfall and sunshine, the temperature, elevation, and landform may be the key causes of genetic variation. In addition, the AMOVA results showed that rich diversity existed in each population, and this was suggested to be the effect of rice cultivar on the population. Wang et al. [25] used RAPD and SNP markers to analyze the genetic diversity of *U. virens* strains isolated from two different rice cultivars in the same area and found that rice variety played a crucial role in the genetic variation of *U. virens*.

In previous studies, the *U. virens* strains were classified into two genetic groups by the tool STRUCTURE [10,26,52]. However, in this study, 221 isolates of *U. virens* from 13 geographical populations were classified into five genetic subgroups. This result suggested a certain level of genetic diversity among different strains in southwest China compared with most of the other regions due to the complexity of the geographical environment. Fang et al. [27] also suggested that the elevation and landform may contribute to the rich genetic diversity of *U. virens* in Sichuan–Chongqing due to climatic conditions. Zhang et al. [50] found that the genetic diversity of *U. virens* was richer in the mountainous and hilly ecozones with complex geographical and climatic conditions than in the plain ecozones. Moreover, the results of population structure were basically consistent with the UPGMA dendrogram of 13 geographical populations and the phylogenetic analysis of 221 *U. virens* strains. In addition, we found that most isolates of *U. virens* were not clustered by geographical origin, but some isolates from different geographical origin clustered together. Therefore, we speculated that gene flow and recombination may exist among the populations. Previous studies have reported gene flow and recombination between different geographical populations [26,27].

In conclusion, the present study provides detailed information about the characteristics of morphology, pathogenicity, mating-type loci distribution, genetic diversity, and population structure of *U. virens* strains from southwest China. The morphological features of strains of *U. virens* collected from 13 rice-planting areas of southwest China showed significant diversity, and the pathogenicity of the fungi showed clear differentiation. These results revealed that the mating types of *U. virens* isolates in southwest China comprised heterothallic and homothallic strains. A total of 221 strains of *U. virens* from 13 geographical populations were divided into five genetic subgroups, indicating genetic variation within or among geographical populations. This comprehensive understanding of the population structure and diversity of *U. virens* will provide useful information for the comprehensive control of rice false smut disease.

## Figures and Tables

**Table 1:** Different strains of *Ustilaginoidea virens* collected from southwest China in the study.

Isolate	Location	City	Rice Variety/Line	Year of Collection	*Mat1-1-1*	*Mat1-2-1*
BZN1-1	Nanjiang	Bazhong	Deyou4727	2018	-	+
BZN1-2	Nanjiang	Bazhong	Deyou4728	2018	-	+
CDC1	Chongzhou	Chengdu	Gangyou36	2019	+	-
CDC2	Chongzhou	Chengdu	Chuanyou72	2019	+	-
CDC3	Chongzhou	Chengdu	Chuanyou72	2019	-	+
CDC4	Chongzhou	Chengdu	Jingyou727	2019	-	+
CDJ1-1	Jintang	Chengdu	Yixiangyou2115	2017	-	+
CDJ1-2	Jintang	Chengdu	Yixiangyou2115	2017	-	+
CDM1-1	Pengzhou Mengyang	Chengdu	Leyou891	2019	+	-
CDM1-2	Pengzhou Mengyang	Chengdu	Leyou891	2019	+	+
CDM1-3	Pengzhou Mengyang	Chengdu	Leyou891	2019	+	+
CDM1-4	Pengzhou Mengyang	Chengdu	Leyou891	2019	-	+
CDP1-1	Pixian	Chengdu	Wanyou66	2017	-	+
CDP1-2	Pixian	Chengdu	Wanyou66	2017	+	+
CDP2	Pixian	Chengdu	Rongdao415	2019	-	+
CDP3	Pixian	Chengdu	Neixiang8514	2019	+	-
CDP4	Pixian	Chengdu	Rongyou1808	2019	+	-
CDP5	Pixian	Chengdu	Yliangyou1	2019	+	+
CDP6	Pixian	Chengdu	Gang8you316	2019	+	-
CDPJ1	Pujiang	Chengdu	Fuyou838	2017	+	-
CDPJ2	Pujiang	Chengdu	Mianyou5323	2017	-	+
CDPJ3	Pujiang	Chengdu	Gangyou900	2017	-	+
CDPJ4	Pujiang	Chengdu	Gangyou900	2019	-	+
CDPJ5	Pujiang	Chengdu	Gangyou900	2019	+	-
CDPJ6	Pujiang	Chengdu	Mianyou5323	2017	+	-
CDPJ7	Pujiang	Chengdu	Fuyou838	2017	+	+
CDPJ8	Pujiang	Chengdu	Fuyou838	2019	+	-
CDPJ9	Pujiang	Chengdu	Gangyou900	2018	+	-
CDQ1-1	Qionglai	Chengdu	Nei5you16	2017	-	+
CDQ1-2	Qionglai	Chengdu	Jing1youhuazeng	2019	-	+
CDQ2-1	Qionglai	Chengdu	Nei5you16	2017	+	-
CDQ2-2	Qionglai	Chengdu	Nei5you16	2019	-	+
CDQ2-3	Qionglai	Chengdu	Nei5you16	2017	+	-
CDQ2-4	Qionglai	Chengdu	Jing1youhuazeng	2017	+	-
CDQ3-1	Qionglai	Chengdu	Jing1youhuazeng	2019	+	+
CDQ3-2	Qionglai	Chengdu	Jing1youhuazeng	2019	-	+
CDX1-1	Xindu	Chengdu	Guichao 2	2017	+	-
CDX1-2	Xindu	Chengdu	Jiangyou527	2019	-	+
CDX2-1-1	Xindu	Chengdu	Guichao 2	2017	-	+
CDX2-1-2	Xindu	Chengdu	Guichao 2	2019	-	+
CDX2-2-1	Xindu	Chengdu	Guichao 2	2017	+	+
CDX3-1	Xindu	Chengdu	Jiangyou527	2017	-	+
CDX3-2	Xindu	Chengdu	Jiangyou527	2017	+	-
CDX3-3	Xindu	Chengdu	Jiangyou527	2017	+	-
CDX4-1	Xindu	Chengdu	Guichao 2	2017	+	-
CDX4-2	Xindu	Chengdu	Jiangyou527	2017	+	-
CDX5-1	Xindu	Chengdu	Fyou399	2017	-	+
CDX5-2	Xindu	Chengdu	Fyou399	2017	-	+
CDX5-3	Xindu	Chengdu	Fyou399	2019	+	-
CDX5-4	Xindu	Chengdu	Fyou399	2019	-	+
CDX6-1	Xindu	Chengdu	Jiangyou527	2019	+	-
CDX7-1	Xindu	Chengdu	Daosongjing9	2018	-	+
CDX8-1-1	Xindu	Chengdu	Huaxiang7A	2018	-	+
CDX8-1-2	Xindu	Chengdu	Huaxiang7A	2018	-	+
CDX8-1-3	Xindu	Chengdu	Huaxiang7A	2018	-	+
CDX8-1-4	Xindu	Chengdu	Huaxiang7A	2018	-	+
CDX8-1-5	Xindu	Chengdu	Huaxiang7A	2018	-	+
CDX8-1-6	Xindu	Chengdu	Huaxiang7A	2018	-	+
CDX8-2-1	Xindu	Chengdu	Huaxiang7A	2018	-	+
CDX8-2-2	Xindu	Chengdu	Huaxiang7A	2018	-	+
CDX8-2-3	Xindu	Chengdu	Huaxiang7A	2018	-	+
CDX8-2-4	Xindu	Chengdu	Huaxiang7A	2018	-	+
CDX8-2-5	Xindu	Chengdu	Huaxiang7A	2018	-	+
CQN1-1-1	Nanan	Chongqing	Neiyou683	2019	-	+
CQN1-1-2	Nanan	Chongqing	Neiyou683	2019	-	+
CQN1-1-3	Nanan	Chongqing	Neiyou683	2019	+	-
CQN1-1-4	Nanan	Chongqing	Neiyou683	2019	+	-
CQN1-1-5	Nanan	Chongqing	Neiyou683	2019	+	-
CQN1-2-1	Nanan	Chongqing	Neiyou683	2019	+	-
CQN1-2-2	Nanan	Chongqing	Neiyou683	2019	+	-
CQN1-2-3	Nanan	Chongqing	Neiyou683	2019	+	-
CQN2-1-1	Nanan	Chongqing	Dyou166	2019	-	+
CQN2-1-2	Nanan	Chongqing	Dyou166	2019	+	+
CQN2-1-3	Nanan	Chongqing	Dyou166	2019	+	+
CQN2-1-4	Nanan	Chongqing	Dyou166	2019	-	+
CQN2-1-5	Nanan	Chongqing	Dyou166	2019	+	-
CQN2-1-6	Nanan	Chongqing	Dyou166	2019	-	+
DY1	Deyang	Deyang	Qianxiangyou677	2018	+	-
DYG1	Guanghan Xigao	Deyang	Daosongjing9	2017	-	+
DYG2	Guanghan Xigao	Deyang	Daosongjing9	2019	-	+
DYG3	Guanghan Xigao	Deyang	Daosongjing9	2017	+	+
DYG4	Guanghan Xigao	Deyang	Daosongjing9	2019	+	-
DYG5	Guanghan Xigao	Deyang	Daosongjing9	2019	-	+
DYG6-1-1	Guanghan	Deyang	Qianxiangyou677	2019	-	+
DYG6-1-2	Guanghan	Deyang	Jiangyou126	2019	-	+
DYG6-1-3	Guanghan	Deyang	Jiangyou126	2019	-	+
DYG6-1-4	Guanghan	Deyang	Jiangyou126	2019	-	+
DYG6-1-5	Guanghan	Deyang	Jiangyou126	2019	-	+
DYG6-1-6	Guanghan	Deyang	Jiangyou126	2019	-	+
DYG6-2-1	Guanghan	Deyang	Jiangyou126	2019	-	+
DYG6-2-2	Guanghan	Deyang	Jiangyou126	2019	-	+
DYG6-2-3	Guanghan	Deyang	Jiangyou126	2019	-	+
DYG6-2-4	Guanghan	Deyang	Jiangyou126	2019	-	+
DYL1-1	Luojiang	Deyang	Rongyou 908	2019	-	+
DYL1-2	Luojiang	Deyang	Rongyou 908	2019	-	+
DYM1-1	Mianzhu	Deyang	Gangyou188	2018	-	+
DYM2-1-1	Mianzhu	Deyang	Zhongliangyou	2018	-	+
DYM2-1-2	Mianzhu	Deyang	Gangyou188	2018	-	+
DYM2-1-3	Mianzhu	Deyang	Gangyou188	2018	-	+
DYM2-1-4	Mianzhu	Deyang	Gangyou188	2018	-	+
DYM2-1-5	Mianzhu	Deyang	Mianyou5323	2018	-	+
DYM2-2-1	Mianzhu	Deyang	Mianyou5323	2018	-	+
DYM2-2-2	Mianzhu	Deyang	Mianyou5323	2018	-	+
DYM2-2-3	Mianzhu	Deyang	Mianyou5323	2018	-	+
DYM2-2-4	Mianzhu	Deyang	Mianyou5323	2018	-	+
DYM2-2-5	Mianzhu	Deyang	Mianyou5323	2018	-	+
DYM2-2-6	Mianzhu	Deyang	Mianyou5323	2018	-	+
DYM2-3-1	Mianzhu	Deyang	Mianyou5323	2018	+	-
DYM2-3-2	Mianzhu	Deyang	Mianyou5323	2018	+	-
DYM2-3-3	Mianzhu	Deyang	Mianyou5323	2018	+	-
DYM2-3-4	Mianzhu	Deyang	Mianyou5323	2018	+	-
DYM2-3-5	Mianzhu	Deyang	Mianyou5323	2018	+	-
DYM2-3-6	Mianzhu	Deyang	Mianyou5323	2018	+	-
DYS1	Shifang	Deyang	Yliangyou1	2018	-	+
GYJ1-1	Jingzhu	Guiyang	Quanxiangyou191	2019	-	+
GYJ1-2	Jingzhu	Guiyang	Quanxiangyou191	2019	-	+
GYJ1-3	Jingzhu	Guiyang	Quanxiangyou191	2019	-	+
GYJ1-4	Jingzhu	Guiyang	Quanxiangyou191	2019	-	+
GYJ1-5	Jingzhu	Guiyang	Quanxiangyou191	2019	-	+
GYJ1-6	Jingzhu	Guiyang	Quanxiangyou191	2019	-	+
LSS1	Shuangxi	Leshan	Gangyou900	2019	-	+
LSS2	Shuangxi	Leshan	Gangyou900	2019	-	+
LSS3	Shuangxi	Leshan	Gangyou900	2019	-	+
LZB1-4	Gulin	Luzhou	Fuyou838	2019	-	+
LZB2-1	Baijie	Luzhou	Rongyou87	2019	-	+
LZB2-2	Baijie	Luzhou	Fuyou838	2019	-	+
LZD1	Danlin	Luzhou	Chuanyou72	2018	-	+
LZD2	Danlin	Luzhou	Jingyou727	2018	+	+
LZG1-1	Gulin	Luzhou	Rongyou87	2017	-	+
LZG1-2	Gulin	Luzhou	Rongyou87	2018	+	+
LZG1-3	Gulin	Luzhou	Rongyou87	2019	+	-
LZG1-5	Gulin	Luzhou	Fuyou838	2018	+	+
LZL4-1	Luxian	Luzhou	Fuyou838	2018	+	-
LZN3-1	Naxi	Luzhou	Fuyou838	2017	-	+
KMJ1-1	Jinning	Kunming	Yunda107	2018	-	+
KMJ2-1	Jinning	Kunming	Yunda107	2019	+	-
KMJ3-1	Jinning	Kunming	Yunliangyou501	2018	-	+
KMJ4-1	Jinning	Kunming	Yunliangyou501	2017	+	-
KMJ2-2	Jinning	Kunming	Yunda107	2019	+	-
KMJ1-2	Jinning	Kunming	Yunda107	2019	+	-
KMJ3-2	Jinning	Kunming	Yunliangyou501	2019	+	-
KMJ3-3	Jinning	Kunming	Yunliangyou501	2019	-	+
NCY1	Yingshan	Nanchong	Dexiangyou146	2019	-	+
NCY2	Yingshan	Nanchong	Dexiangyou146	2019	+	-
NCY3	Yingshan	Nanchong	Yixiang2079	2019	+	-
NCY4	Yingshan	Nanchong	Yixiang2079	2018	-	+
LJY1	Yongning	Lijiang	Ligeng15	2019	+	+
LJY2	Yongning	Lijiang	Ligeng15	2019	+	-
LJY3	Yongning	Lijiang	Ligeng9	2019	+	+
LJY4	Yongning	Lijiang	Ligeng15	2019	-	+
LJY5	Yongning	Lijiang	Ligeng9	2019	+	-
LJY6	Yongning	Lijiang	Ligeng9	2019	+	-
LJY7	Yongning	Lijiang	Ligeng9	2019	+	-
YAZ1	Zhongli	Yaan	Fuyou838	2018	-	+
YAZ2	Zhongli	Yaan	Gangyou3551	2019	+	+
YAZ3	Zhongli	Yaan	Fuyou838	2019	-	+
YAZ4	Zhongli	Yaan	Yixiang1577	2019	-	+
YAZ5	Zhongli	Yaan	Fuyou838	2018	+	+
YAZ6	Zhongli	Yaan	Fuyou838	2018	-	+
YAZ7	Zhongli	Yaan	Fuyou1	2018	-	+
YBN1	Nanxi	Yibin	Yliangyou1146	2019	-	+
YBN2	Nanxi	Yibin	Yliangyou1147	2019	+	-
YBN3	Nanxi	Yibin	Suyou727	2019	+	-
YBN4	Nanxi	Yibin	Suyou727	2019	+	-
YBN5	Nanxi	Yibin	Suyou727	2019	+	-
ZYX1-1-1	Meitan Xinglong	Zunyi	Zhongkexilu 1	2019	-	+
ZYX1-1-2	Meitan Xinglong	Zunyi	Zhongkexilu 1	2019	-	+
ZYX1-1-3	Meitan Xinglong	Zunyi	Zhongkexilu 1	2019	-	+
ZYX1-1-4	Meitan Xinglong	Zunyi	Zhongkexilu 1	2019	-	+
ZYX1-1-5	Meitan Xinglong	Zunyi	Zhongkexilu 1	2019	-	+
ZYX1-1-6	Meitan Xinglong	Zunyi	Zhongkexilu 1	2019	-	+
ZYX1-2-1	Meitan Xinglong	Zunyi	Zhongkexilu 1	2019	+	-
ZYX1-2-2	Meitan Xinglong	Zunyi	Zhongkexilu 1	2019	+	-
ZYX2-1-1	Meitan Xinglong	Zunyi	Chengyouyuenongsimiao	2019	-	+
ZYX2-1-2	Meitan Xinglong	Zunyi	Chengyouyuenongsimiao	2019	-	+
ZYX2-1-3	Meitan Xinglong	Zunyi	Chengyouyuenongsimiao	2019	-	+
ZYX2-1-4	Meitan Xinglong	Zunyi	Chengyouyuenongsimiao	2019	-	+
ZYX2-1-5	Meitan Xinglong	Zunyi	Chengyouyuenongsimiao	2019	-	+
ZYX2-1-6	Meitan Xinglong	Zunyi	Chengyouyuenongsimiao	2019	-	+
ZYX2-2-1	Meitan Xinglong	Zunyi	Chengyouyuenongsimiao	2019	-	+
ZYX2-2-2	Meitan Xinglong	Zunyi	Chengyouyuenongsimiao	2019	-	+
ZYX2-2-3	Meitan Xinglong	Zunyi	Chengyouyuenongsimiao	2019	-	+
ZYX2-2-4	Meitan Xinglong	Zunyi	Chengyouyuenongsimiao	2019	-	+
ZYX2-2-5	Meitan Xinglong	Zunyi	Chengyouyuenongsimiao	2019	-	+
ZYX2-3-1	Meitan Xinglong	Zunyi	Chengyouyuenongsimiao	2019	-	+
ZYX2-3-2	Meitan Xinglong	Zunyi	Chengyouyuenongsimiao	2019	-	+
ZYX3-1-1	Meitan Xinglong	Zunyi	Liangyou 198	2019	-	+
ZYX3-1-2	Meitan Xinglong	Zunyi	Liangyou 198	2019	-	+
ZYX3-1-3	Meitan Xinglong	Zunyi	Liangyou 198	2019	-	+
ZYX3-1-4	Meitan Xinglong	Zunyi	Liangyou 198	2019	-	+
ZYX3-1-5	Meitan Xinglong	Zunyi	Liangyou 198	2019	-	+
ZYX3-1-6	Meitan Xinglong	Zunyi	Liangyou 198	2019	-	+
ZYX4-1-1	Meitan Xinglong	Zunyi	Jiangliangyou 198	2019	-	+
ZYX4-1-2	Meitan Xinglong	Zunyi	Jiangliangyou 198	2019	-	+
ZYX4-1-3	Meitan Xinglong	Zunyi	Jiangliangyou 198	2019	-	+
ZYX4-1-4	Meitan Xinglong	Zunyi	Jiangliangyou 198	2019	-	+
ZYX4-1-5	Meitan Xinglong	Zunyi	Jiangliangyou 198	2019	-	+
ZYX4-1-6	Meitan Xinglong	Zunyi	Jiangliangyou 198	2019	-	+
ZYX5-1-1	Meitan Xinglong	Zunyi	Gongliangyou 23	2019	+	-
ZYX5-1-2	Meitan Xinglong	Zunyi	Gongliangyou 23	2019	+	-
ZYX5-1-3	Meitan Xinglong	Zunyi	Gongliangyou 23	2019	+	-
ZYY1-1	Meitan Yongxing	Zunyi	Quanxiangyou 191	2019	-	+
ZYY1-2	Meitan Yongxing	Zunyi	Quanxiangyou 191	2019	-	+
ZYY1-3	Meitan Yongxing	Zunyi	Quanxiangyou 191	2019	+	-
ZYY1-4	Meitan Yongxing	Zunyi	Quanxiangyou 191	2019	+	-
ZYY1-5	Meitan Yongxing	Zunyi	Quanxiangyou 191	2019	+	-
ZYY1-6	Meitan Yongxing	Zunyi	Quanxiangyou 191	2019	+	-
ZYY2-1	Meitan Yongxing	Zunyi	Lixiangyouxiang 22	2019	-	+
ZYY2-2	Meitan Yongxing	Zunyi	Lixiangyouxiang 22	2019	-	+
ZYY2-3	Meitan Yongxing	Zunyi	Lixiangyouxiang 22	2019	-	+
ZYY2-4	Meitan Yongxing	Zunyi	Lixiangyouxiang 22	2019	-	+
ZYY2-5	Meitan Yongxing	Zunyi	Lixiangyouxiang 22	2019	-	+
ZYY2-6	Meitan Yongxing	Zunyi	Lixiangyouxiang 22	2019	-	+
ZYY2-7	Meitan Yongxing	Zunyi	Lixiangyouxiang 22	2019	-	+
ZYY3-1	Meitan Yongxing	Zunyi	Liangyoufeibo 1	2019	-	+
ZYY3-2	Meitan Yongxing	Zunyi	Liangyoufeibo 1	2019	-	+
ZYY3-3	Meitan Yongxing	Zunyi	Liangyoufeibo 1	2019	-	+
ZYY3-4	Meitan Yongxing	Zunyi	Liangyoufeibo 1	2019	-	+
ZYY3-5	Meitan Yongxing	Zunyi	Liangyoufeibo 1	2019	-	+
ZYY3-6	Meitan Yongxing	Zunyi	Liangyoufeibo 1	2019	-	+
ZYY3-7	Meitan Yongxing	Zunyi	Liangyoufeibo 1	2019	-	+

**Table 2:** Different primers used in the present study.

Genes	Primers	Primer Sequence (5′-3′)	Tm (°C)
*MAT1 locus*	MAT1-1F	GAAGTCGTATGCGTGCGAC	60
	MAT1-1R	CTTGTTCCACAGGGTGGTCA	
	MAT1-2F	CAATCTGCGCTTGGGTGTTC	60
	MAT1-2R	GGAGCGACATAATACCGTCA	
SNP	SNP1F	GGTCGGATACTCGGTGCC	56
	SNP1R	CGCTTAGGGCATCTTTCAC	
	SNP2F	GGTTCCGCTAGGGGCGATTG	56
	SNP2R	TGACGGGGGCGTAGTAAGTTT	
	SNP3F	TTGGCGGAGGAGATCAGGGTG	56
	SNP3R	TGCTGGTGGGAGGCGTTGA	
SSR	RM211F	AATGACGGGCAAGACAAATC	60
	RM211R	TACAACGCCAGCGTTATCTG	
	RM318 F	GGTATTTCGGAAGAATGGCA	60
	RM318 R	ACGGCAGCTTTTAGACATGG	
	RM403F	ATCATGACTGGACCTGAGGC	60
	RM403R	CTGAAACGGTGAACGAGACA	
	RM415F	ACTCGACCTGTCTTGCGACT	60
	RM415R	CATTTGCGGTCTTTTGCTTT	
	RM414 F	TTGTTAGCTCGACGGGTTCT	60
	RM414 R	TGCGGTACGTACTTGTGCTC	
	RM509F	CCGGAGTACCCAGATGCTTA	60
	RM509R	GGAGAAGGTTAAGGTTGGGG	
	RM522 F	AGGATGAGCAAGTCGCAGAT	60
	RM522R	GCTAGGAGCCCTTGGAATTT	
	RM621F	TCGTTATTGAGTCCCGAAGC	60
	RM621R	TGGACGTGAACAACAAGGAG	
	RM523F	TTTCAGCTGCACAACCAAAG	60
	RM523R	GATTCTTCGACTACGGCTGC	

**Table 3:** Polymorphism of SSR primer amplification bands of *Ustilaginoidea virens* isolates.

Primers	Number of Amplified Bands	Number of Polymorphic Bands	Percentage of Polymorphic Bands (%)
RM211	8	6	75.00
RM318	5	3	60.00
RM403	4	3	75.00
RM415	6	4	66.67
RM414	3	2	66.67
RM509	3	1	33.33
RM522	5	3	60.00
RM621	3	2	66.67
RM523	3	1	33.33
Total	40	25	62.50

**Table 4:** Genetic diversity of isolates from different fields in southwest China.

Population	No. of Isolates	Polymorphic Loci (%)	No. of Different Alleles (*Na*)	No. of Effective Alleles (*Ne*)	Nei’s Locus Diversity (*H*)	Shannon’s Index (*I*)
BZ	2	53.08	1.1034	1.0560	0.1701	0.2543
CD	61	61.54	1.4921	1.1891	0.1823	0.2815
CQ	14	59.54	1.4283	1.2921	0.1734	0.2713
DY	37	68.23	1.5020	1.2410	0.1943	0.2955
GY	6	84.62	1.6802	1.3714	0.2794	0.3757
KM	8	80.62	1.6520	1.2385	0.2618	0.3639
LS	3	78.92	1.5706	1.3429	0.2521	0.3524
LJ	7	76.92	1.6109	1.3691	0.2480	0.3304
LZ	11	57.08	1.2063	1.1723	0.1765	0.2736
NC	4	85.67	1.8165	1.4017	0.2893	0.3856
YA	7	72.91	1.5563	1.3301	0.2296	0.3116
YB	5	69.23	1.5515	1.2650	0.2018	0.2998
ZY	56	66.54	1.5054	1.2012	0.1902	0.2925
Total	221	86.13	1.8751	1.4231	0.2994	0.4016

## Data Availability

All the data are present in the manuscript.

## Supplementary Materials

The following supporting information can be downloaded at https://www.mdpi.com/article/10.3390/jof8111204/s1, Figure S1: Dendrogram constructed with the UPGMA clustering method for isolates of *Ustilaginoidea virens* based on nine SSR primers. Five groups were divided at 0.72 genetic distance level, and most isolates were in groups I and IV., Figure S2: Dendrogram of isolates from Deyang and Zunyi by SSR analysis. Figure S3: Phylogenetic analysis based on SNP sequence at individual marker (A–C) or the combined SNP sequence at three markers (D). NJ phylogenetic analyses were conducted based on DNA sequences at marker 1 (A), marker 2 (B), and marker 3 (C) and the combined sequences at three markers (D). Nodal support values for NJ are given for branches receiving more than 70% support. Groups were named with marker names and Roman numerals. Figure S4: Phylogenetic analysis of isolates from Deyang and Zunyi by SNP analysis. NJ phylogenetic analyses were conducted based on the combined sequences at three markers. Nodal support values for NJ are given for branches receiving more than 70% support. Figure S5: Analysis of molecular variance of 221 isolates of *Ustilaginoidea virens* in 13 populations from southwest China. Table S1: Variability in morphology, pathogenicity, and mating type among the different isolates of *Ustilaginoidea virens*. Table S2: Pairwise *F_ST_* values between different populations.
