# Supplementary figures and images for: Diversity Analysis of the Rice False Smut Pathogen Ustilaginoidea virens in Southwest China
Source: J Fungi (Basel). 2022 Nov 15;8(11):1204. doi: 10.3390/jof8111204 (PMC9694781; doi:10.3390/jof8111204)

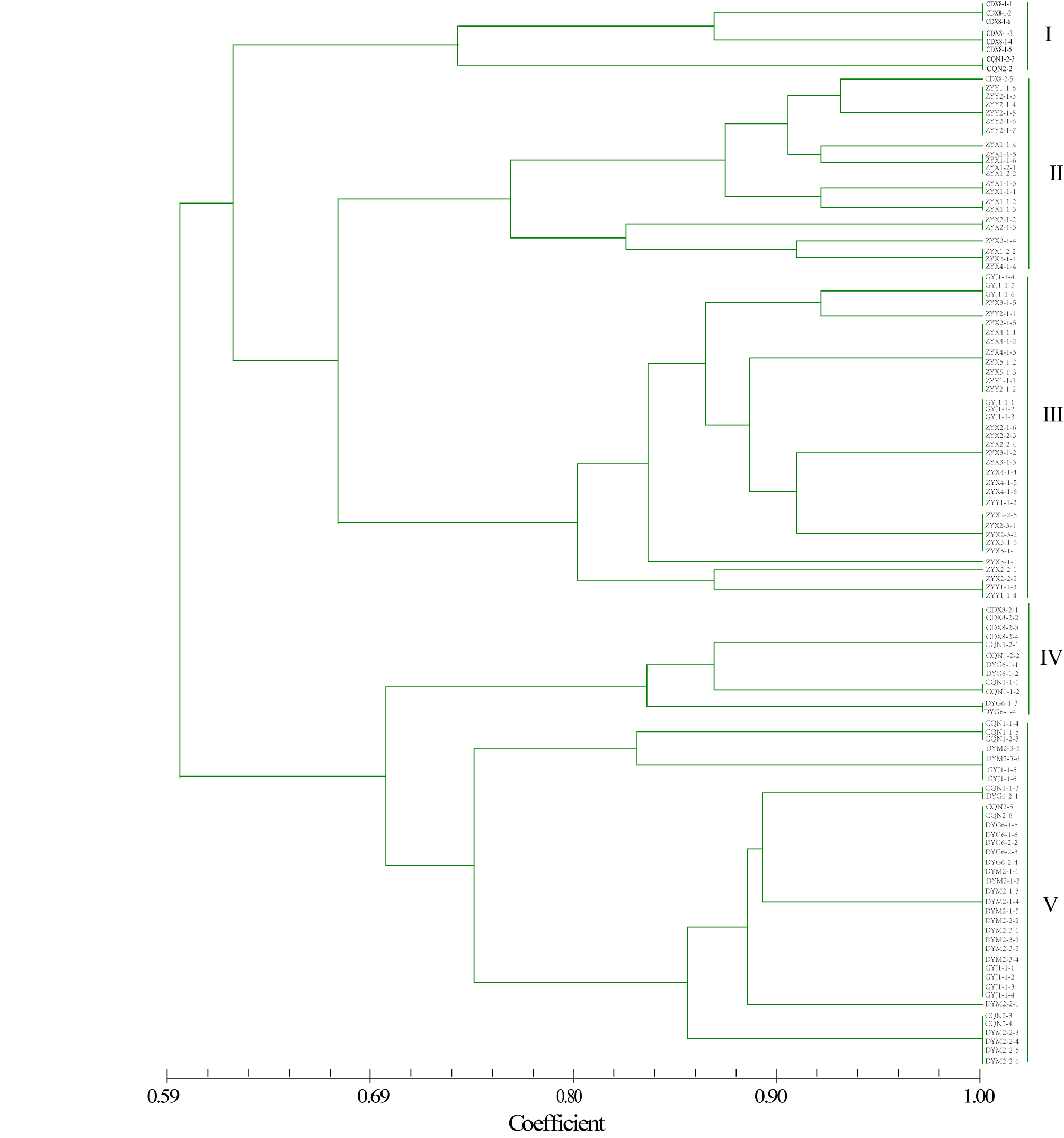

Supplement: Supplementary file 1 [file jof-08-01204-s001.zip › Supplementary Figure S1.tif]

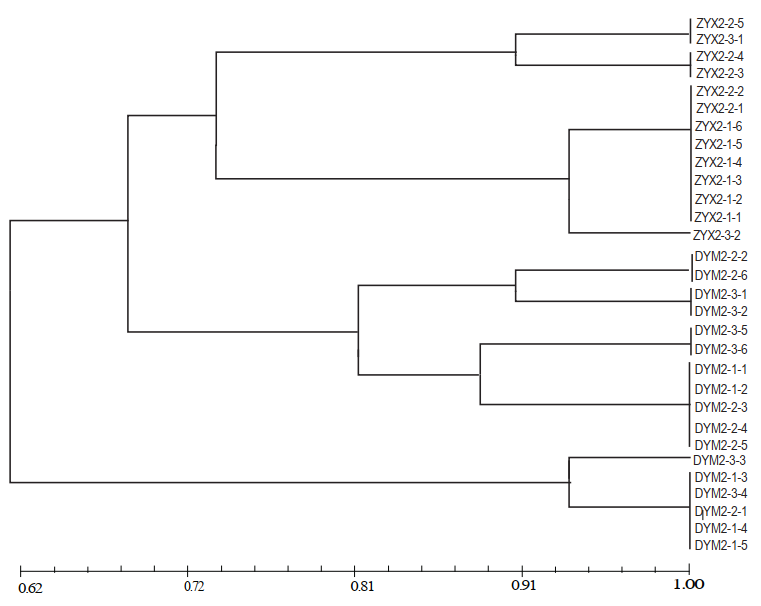

Supplement: Supplementary file 1 [file jof-08-01204-s001.zip › Supplementary Figure S2.jpg]

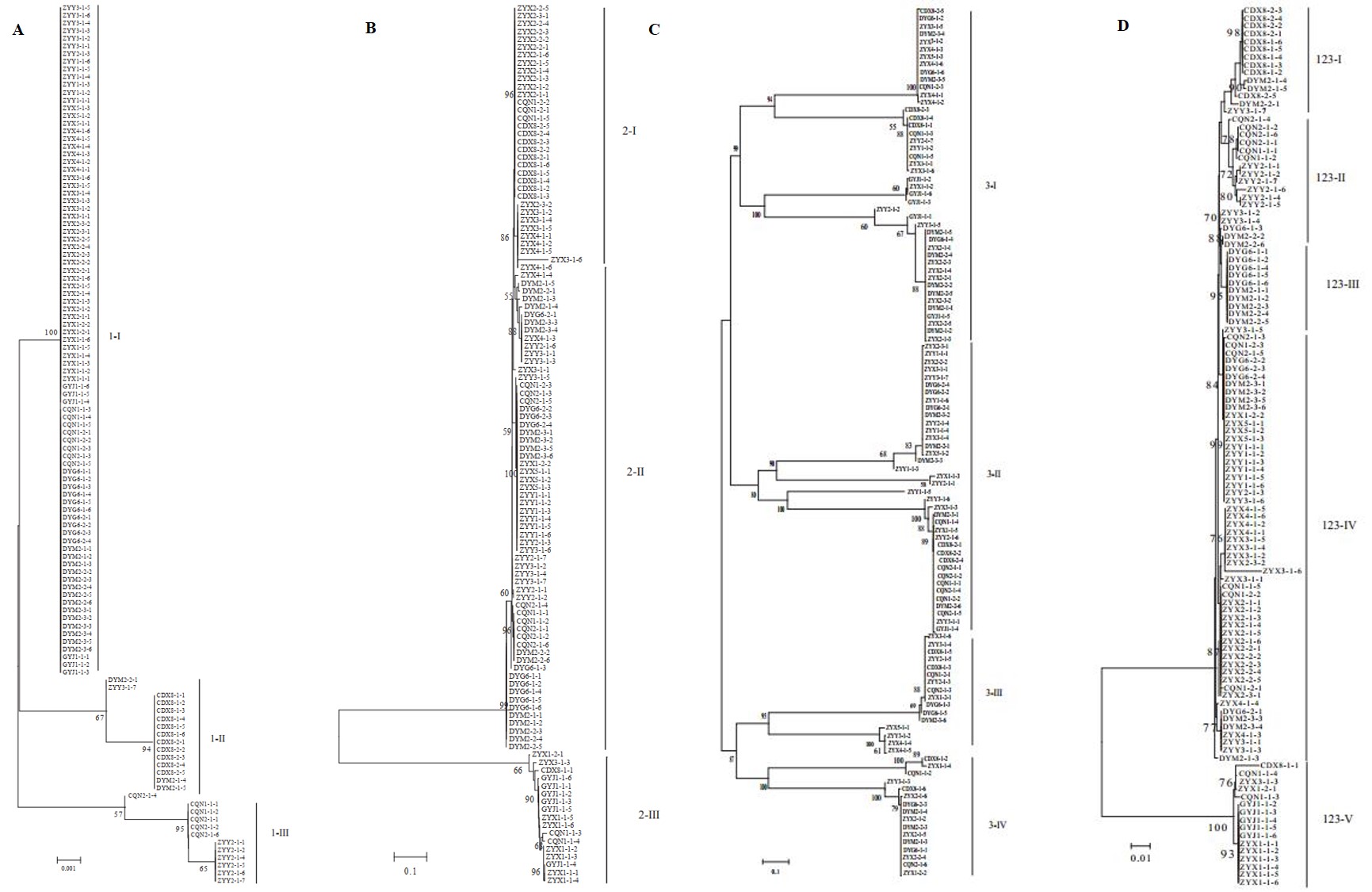

Supplement: Supplementary file 1 [file jof-08-01204-s001.zip › Supplementary Figure S3.jpg]

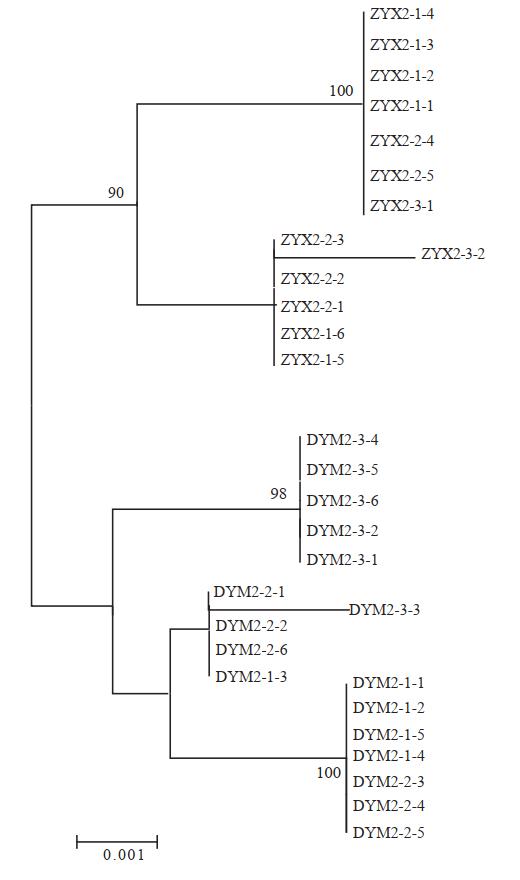

Supplement: Supplementary file 1 [file jof-08-01204-s001.zip › Supplementary Figure S4.jpg]

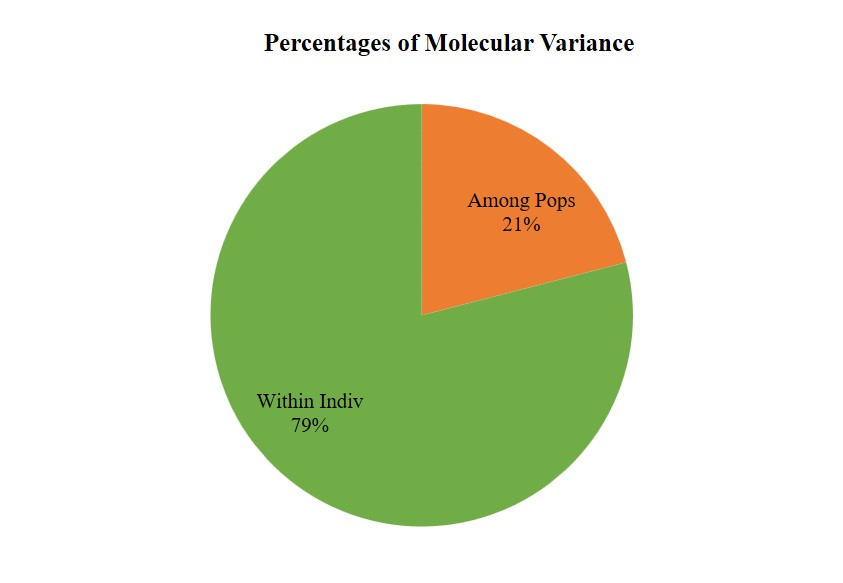

Supplement: Supplementary file 1 [file jof-08-01204-s001.zip › Supplementary Figure S5.jpg]
